# Effect of Orthostasis on Endothelial Function: A Gender Comparative Study

**DOI:** 10.1371/journal.pone.0071655

**Published:** 2013-08-02

**Authors:** Nandu Goswami, Paavan Gorur, Ulrike Pilsl, Bond Anyaehie, David A. Green, Alexander I. Bondarenko, Andreas Roessler, Helmut G. Hinghofer-Szalkay

**Affiliations:** 1 Institute of Physiology, Center of Physiological Medicine, Medical University of Graz, Graz, Austria; 2 Centre of Human and Aerospace Physiological Sciences, King’s College London, London, United Kingdom; 3 Department of Anatomy, Medical University of Graz, Graz, Austria; 4 Department of Physiology, College of Medicine, University of Nigeria, Enugu Campus, Enugu, Nigeria; 5 AA Bogomoletz Inst Physiol, Circulatory Physiol Department, Kiev, Ukrainian State, Ukraine; Universidade Federal do Rio de Janeiro, Brazil

## Abstract

As the vascular endothelium has multiple functions, including regulation of vascular tone, it may play a role in the pathophysiology of orthostatic intolerance. We investigated the effect of orthostasis on endothelial function using EndoPAT®, a non-invasive and user-independent method, and across gender. As sex steroid hormones are known to affect endothelial function, this study examined the potential effect of these hormones on the endothelial response to orthostasis by including females at different phases of the menstrual cycle (follicular and luteal—where the hormone balance differs), and females taking an oral contraceptive. A total of 31 subjects took part in this study (11 males, 11 females having normal menstrual cycles and 9 females taking oral contraceptive). Each subject made two visits for testing; in the case of females having normal menstrual cycles the first session was conducted either 1–7 (follicular) or 14–21 days (luteal) after the start of menstruation, and the second session two weeks later, i.e., during the other phase, respectively. Endothelial function was assessed at baseline and following a 20-min orthostatic challenge (active standing). The EndoPAT® index increased from 1.71 ± 0.09 (mean ± SEM) at baseline to 2.07 ± 0.09 following orthostasis in females (p<0.001). In males, the index increased from 1.60 ± 0.08 to 1.94 ± 0.13 following orthostasis (p<0.001). There were no significant differences, however, in the endothelial response to orthostasis between females and males, menstrual cycle phases and the usage of oral contraceptive. Our results suggest an increased vasodilatatory endothelial response following orthostasis in both females and males. The effect of gender and sex hormones on the endothelial response to orthostasis appears limited. Further studies are needed to determine the potential role of this post orthostasis endothelial response in the pathophysiology of orthostatic intolerance.

## Introduction

Adoption of an upright posture (relative to gravity) can induce a drop in arterial blood pressure (orthostasis). Failure to maintain mean arterial blood pressure can lead to reduced cerebral blood flow, which can precipitate dizziness or even syncope in susceptible (orthostatic intolerant) individuals, and astronauts returning to Earth. Numerous mechanisms appear to underlie orthostatic intolerance (see 1). For instance, returning astronauts appear to a have reduced plasma volume, possibly secondary to the cephalad shift of fluid associated with microgravity exposure including reduced baroreflex sensitivity [2]. Cardiac muscle atrophy has also been observed in astronauts on microgravity exposure [3]. There has been limited study, however, of the vascular endothelium and in particular its potential role in the pathophysiology of orthostatic intolerance [4].

Mechanical stress of the endothelium has been associated with the release of numerous (e.g. including nitric oxide, NO) vasoactive substances [5,6]. Shear stress provided by an orthostatic challenge, 20 min of active standing lead to increases in (brachial artery) flow mediated dilatation [7,8]. However, whilst assessment of brachial artery flow mediated dilatation (FMD) is considered the gold standard [9,10], it is highly operator dependent.

**Figure 1:**
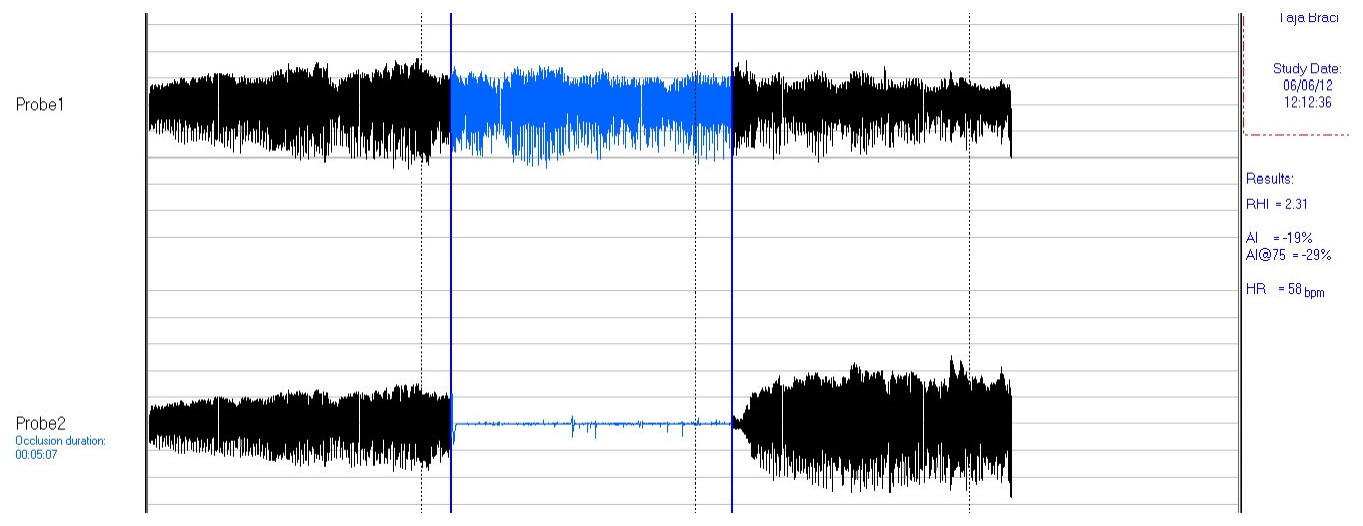
Typical PAT trace recorded on EndoPAT software. Suprasystolic pressure of the blood pressure cuff was confirmed by a flat line (seen in bottom trace) instead of pulsatile flow during occlusion. The EndoPAT index was calculated by the software and represents the ratio of the post occlusion PAT signal to the baseline signal.

A novel non-invasive and user-independent method of assessing endothelial function via (finger) peripheral arterial tonometry (PAT) has been proposed where pulse wave amplitude (PWA) changes are significantly associated with FMD [11]. The EndoPAT2000 (Itamar Medical Ltd, Caesarea, Israel) provides clinicians with reliable and reproducible assessments of endothelial function in 15 min. It has been FDA approved and is also CE marked. Using modified plethysmographic biosensors, the PAT signal is measured from the fingertip by measuring arterial pulsatile volume changes. As in brachial artery flow mediated dilatation assessment, a cuff is inflated around the upper arm to obstruct flow and released. The surge of blood flow causes endothelial dependent FMD (again, a NO mediated response). The dilatation, manifested as a reactive hyperaemia, is shown as an increase in the PAT signal amplitude (Figure 1, below). The EndoPAT^®^ uses beat-to-beat plethysmographic assessment of PWA to calculate the reactive hyperaemic index (RHI); the ratio of digital pulse volume during reactive hyperaemia and the baseline [12]. Computer software then produces an EndoPAT index, a functional score of endothelial function (detailed in methodology section). This study is first to investigate endothelial function in response to orthostatic challenge with the EndoPAT^®^.

Gender appears a significant factor in determining an individual’s susceptibility to orthostatic intolerance [13,14]. A study from 25 female and 140 male astronauts after short duration space flight (5–16 days) showed presyncopal events occurring in 28% of females and 7% of males, associated with low vascular resistance [15]. It appears, therefore, that females are more orthostatically labile [16]. This increased predisposition may be, in part, due to the hormonal differences between males and females. Studies have also shown that vasodilatory endothelial function is increased in periods of the menstrual cycle where estrogen is raised i.e. end of follicular and mid-luteal phase [17]. However, the influence of menstrual cycle phase on the endothelial response to orthostasis is unknown. Furthermore, vasodilatory endothelial function, may be particularly affected by exogenous sex hormones provided by the oral contraceptive pill (OCP) [18,19].

This study, therefore, utilised the EndoPAT^®^, to specifically investigate the: 1) effect of orthostatic challenge on endothelial function; 2) whether it differs according to gender; 3) and if sex steroids modify such an effect, by studying responses in a) females in the follicular and luteal phase of the menstrual cycle, where estrogen levels differ and b) females on the oral contraceptive pill (OCP).

## Methodology

### Subjects

31 (11 males and 20 females [9 on the OCP]) healthy (table **1**), un-trained, non-obese, non-smoking subjects gave written and verbal informed consent to participate in the study that received approval from the Ethics Committee of the Medical University of Graz. The informed consent form was submitted to the Ethics Committee along with other relevant documents when applying for the Ethics approval. Once signed by the subjects, the informed consent were kept by at the Insitute of physiology, where the experiments took place.

**Table 1:** Demographics of the study subjects grouped according to gender and whether taking the oral contraceptive pill (OCP).

	**Males**	**Females not taking OCP**	**Females taking OCP**
**Number (n)**	11	11	9
**Age (years)**	25.3 ± 6.6	27.5 ± 9.6	21.6 ± 2.4
**Weight (kg)**	71.2 ± 7.8	63.6 ± 7.0	66.8 ± 9.0
**Height (m)**	1.77 ± 0.06	1.66 ± 0.04	1.70 ± 0.06
**BMI (kg/m^2^)**	22.7 ± 1.9	23.0 ± 2.5	22.7 ± 2.1

Values are Mean ± SD.

Subjects with histories of vasovagal syncope, vertigo and vestibular disturbance as well as cardiovascular, respiratory and neurological diseases were excluded from the study. The subjects were anonymised during data analyses. In addition, data are stored according to the National Data protection data at the institute, where the experiments were done. These data are not publicly deposited.

Each subject attended two experimental sessions at the Institute of Physiology, Medical University of Graz separated by 2 weeks having been instructed to refrain from exercise and caffeine consumption for >12 hours prior to testing. For females not taking the OCP, the first session was conducted in one phase of the menstrual cycle (*follicular*: 1-7 days after the start of menstruation or *luteal*: 14-21 days after the start of menstruation) and the second session two weeks later. Females on the OCP in this study were either on the combined pill or were on Progesterone only pill. Subjects were tested at the same time of day between 10 am -1 pm in both sessions within a quiet darkened room maintained at ~23° C and 55% humidity.

### Protocol

Each subject had an initial (pre) blood pressure measurement made when supine. The recorded individual systolic blood pressure values were used to calculate the suprasysolic values (20 mmHg above the sysolic pressure) to which the sphygmomanometer cuff was inflated (see below). After blood pressure measurements, no measurements were done for 20 min. Then the endothelial function was assessed using EndoPAT^®^ 2000 (Itamar Medical Ltd, Caesarea, Israel). Subjects then performed the 20 min stand test (on a flat posturography plate) during which blood pressure was checked at regular intervals before returning to the supine position where endothelial function and blood pressure were reassessed (post). Data for blood pressure and posturography will be reported elsewhere.

### Endothelial Function Testing

Endothelial function was assessed using the EndoPAT2000^®^ (Itamar Medical Ltd, Caesarea, Israel) with the subject in the supine position and the hands elevated (via a small platform) so the fingers were hanging freely and a plethysmographic biosensor (finger probes) was applied to each index finger connected to the EndoPAT2000^®^ via flexible tubing. A standard blood pressure cuff (DS 400 Aneroid Sphygmomanometer, Hokanson, Bellevue, USA) was placed around the left upper arm.

Following 5-min PAT signal measurement (baseline), the blood pressure cuff was inflated to 20mmHg greater than each subject’s systolic blood pressure (*supra-systolic pressure*), thus occluding forearm blood flow (confirmed by observation of a flat line real-time PAT signal vs. the pulsatile PAT signal seen in the control arm [Figure **1**]). The cuff remained inflated for 5 min after which the PAT signal was measured for a further 5 min period (recovery).

### Orthostatic challenge Test

Orthostatic challenge was generated via an active stand test where subjects stood on a horizontal surface (posturography plate) for a period of 20 min. Each subject was monitored closely for symptoms of presyncope, which would have led to experimental termination. However, no subject experienced presyncopal symptoms and thus all test subjects completed the entire protocol.

### Data Analysis

In addition to real-time viewing of the PAT signal the software generated the EndoPAT/Reactive Hyperemia Index (RHI) which represents a ratio of the average pulse wave amplitude a 210s (baseline) prior to the blood pressure cuff being inflated vs. 60s after the release of the blood pressure cuff (*post*) from both fingers, thereby correcting for any systemic vascular changes resulting from flow occlusion in the measurement arm.

Changes in EndoPAT index are expressed as changes in mean (± SEM). Two factor ANOVA, with a Bonferroni post-hoc test, was used to obtain the effects of both orthostasis and gender on the EndoPAT index. Similar statistical analysis was used for the EndoPAT data from females relevant to phase of the menstrual cycle as well as whether they were taking the pill or not.

## Results

### The Effect of Orthostasis on Endothelial Function

**Figure 2:**
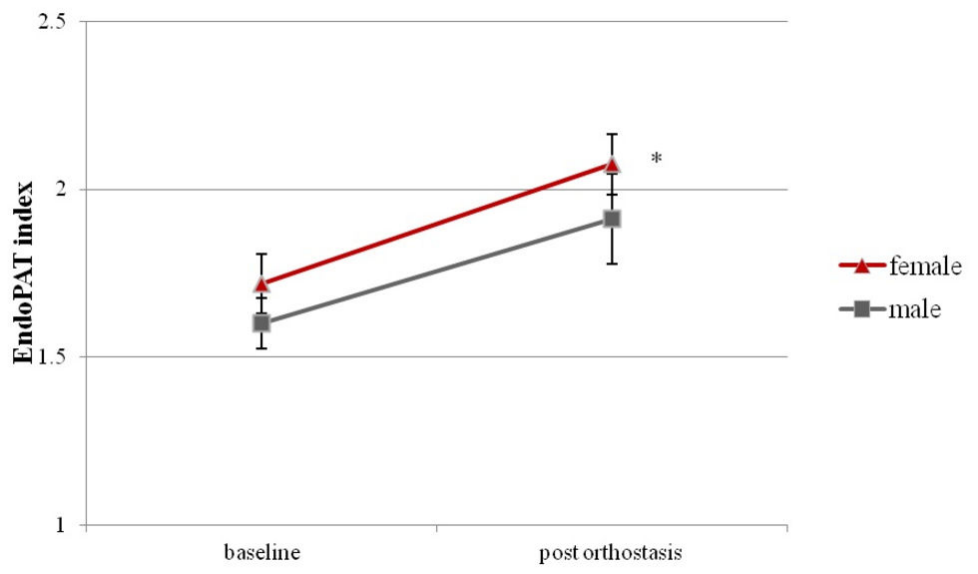
Mean ± SEM EndoPAT index in females and males at baseline and following 20 min of orthostatic challenge. * Indicates significance for change in EndoPAT index from baseline (p<0.001).

The changes in EndoPAT index to orthostasis were measured in 11 males and 20 females, during the two visits (Figure **2**). In females, the baseline EndoPAT index was 1.71 ± 0.09 (mean ± SEM). Twenty min of orthostasis increased the EndoPAT index to 2.07 ± 0.09 (p < 0.001). A statistically significant increase in EndoPAT index was also found in males, with the index rising from 1.60 ± 0.08 at baseline to 1.91 ± 0.13 following orthostasis (p < 0.001).

### Gender Difference in the Effect of Orthostasis on Endothelial Function (Hypothesis #2)

There were no differences in the baseline EndoPAT index readings between females and males. Similarly, there were no significant differences in the post orthostasis EndoPAT RHI values between the sexes (p=0.47).

### The Effect of Menstrual Cycle Phase on Endothelial Response to Orthostasis (Hypothesis #3)

In females the degree of change in EndoPAT RHI post orthostasis was comparable in the two menstrual cycle phases (n = 11) (table **2**). During the follicular phase, the increase in EndoPAT index from baseline to post orthostasis was 0.31 ± 0.15. In the luteal phase, this increase was 0.27 ± 0.33. Statistical analysis, however, revealed no significant differences in the response to orthostasis between menstrual cycle phases (p=0.407).

**Table 2:** Difference in EndoPAT index from baseline post orthostasis for individual female subjects during the follicular and luteal phases of the menstrual cycle.

	Menstrual Cycle Phase	
**Subject**	Follicular	Luteal
**1**	-0.31	0.41
**2**	0.58	0.27
**3**	0.07	0.07
**4**	-0.49	-0.94
**5**	-0.16	0.13
**6**	0.25	1.84
**7**	0.56	-0.08
**8**	1.25	2.39
**9**	0.52	0.28
**10**	0.52	-1.54
**11**	0.59	0.19
**Mean**	**+0.31**	**+0.27**
**SEM**	**0.15**	**0.33**

Values are Mean ± SEM.

### Effect of Oral Contraceptive Pill on Endothelial Responses to Orthostasis (Hypothesis #4)

Altogether nine subjects on the OCP were studied. All of these were on combined OCP or progesterone only pill. Out of these subjects, however, the OCP phase, inferred from the time of their first day of menstrual bleeding, was only available in seven subjects. In these seven subjects (table **3**), the increase in EndoPAT index post orthostasis from baseline in the placebo phase (when no pill or a placebo pill was taken) was 0.15 ± 0.11. In the active phase (when the hormone containing pill was taken), the increase in the index was larger at 0.46 ± 0.21. This increase, however, was not statistically significant (p=0.12).

**Table 3:** Difference in EndoPAT index from baseline post orthostasis for individual female subjects on the OCP during the placebo phase (the one week period when no pill or a placebo pill was taken) and active phase (the period when the hormone containing pill was taken) of the OCP cycle plus Mean ± SEM.

	**Contraceptive Phase**	
**Subject**	Placebo phase	Active phase
**1**	0.41	1.35
**2**	0.55	0.13
**3**	-0.2	-0.31
**4**	-0.18	0.75
**5**	0.16	0.86
**6**	0.31	0.33
**7**	-0.01	0.09
**Mean**	+0.15	+0.46
**SEM**	0.11	0.21

## Discussion

This study shows that 20 min period of orthostatic challenge increases the vasodilator endothelial response, as evidenced by an increased EndoPAT index post orthostasis (confirming hypothesis 1). In addition, the endothelial response to changes of posture from supine to standing was also observed in both females and males. To our knowledge we are not aware of any study that investigated endothelial vascular responses following 20 min of active standing and across gender, using the non-invasive and operator independent EndoPAT®.

The increased vasodilatory response seen post orthostasis represents increased action of NO. One possibility for this increased action is that on standing, the gravitational effects on blood causes pooling in the extremities. This may be seen to increase shear stress on the endothelium, promoting NO release. Studies have also shown an interaction between the sympathetic nervous system and NO action. It is believed that NO may act in modulating sympathetic vasoconstriction, by increasing basal vasodilatory tone [21,22]. The increased vasodilatory endothelial response seen here may represent increased NO production (either to shear stress or to the direct stimulation of NO release by the action of the sympathetic nervous system [23]) thus preventing an exaggerated sympathetic vasoconstrictor response. Our results indicate that the endothelium responds to a 20 min orthostatic challenge. Given its potential role in the modulation of the sympathetic response, alterations in the endothelial response may play a role in in the development of orthostatic intolerance.

Previous studies have looked at the effect of orthostasis on endothelial function. For instance, brachial artery flow mediated dilatation was assessed in 21 males following 60° head up tilt (HUT). The results showed an increase in flow-mediated dilatation in the HUT position compared to baseline [7]. This suggests increased endothelial reactivity following orthostatic challenge. These results were further validated in a study involving normal subjects and hypertensive subjects [8]. The findings of our study are in keeping with these studies. The ability of the EndoPAT^®^ device in showing changes in vasodilatory endothelial function in keeping with previous literature is important. As it is not user dependent, it helps to confirm the response to orthostasis seen before. In addition, given that it has clear advantages in terms of ease of use and non-operator dependence, it may be useful in future studies in this area.

Given the increased susceptibility of females to orthostatic intolerance [14–16], this study examined potential gender differences in the endothelial response to orthostasis. There was no difference found in the response to orthostasis between the sexes. It is possible that the sample size of males included in this study (about half the number of females enrolled in the study) could explain the lack of difference between the sexes. Future studies should examine a larger number of age and health matched subjects from both sexes.

Our study investigated the endothelial response to orthostasis in the follicular and luteal phases of the menstrual cycle. A previous study used brachial artery mediated dilatation on premenopausal women at various stages of the menstrual cycle to assess the effect on endothelial function [24]. Results showed an increase in endothelial function (i.e. FMD) from early to late follicular phase followed by a drop in the early luteal phase. This drop then recovered in the late luteal phase. Similar results have been also been obtained in another study involving premenopausal women and male controls [25]. Here, female subjects were split into three groups – menstrual phase, follicular phase and luteal phase. Results showed statistically significant increases in flow mediated dilatation during the follicular and luteal phase compared to the menstrual phase. Interestingly, the flow mediated dilatation responses in women during the menstrual phase were comparable to the response in the male subjects. In both of these studies, the changes in endothelial function were postulated to be due to the positive effects of estrogen. This would account for the increased flow mediated dilatation seen during the late follicular and late luteal phases, when estrogen levels are raised. Males and the early follicular menstrual phase both represent a lower estrogen state, which may explain the reduced endothelial function. Our results, however, do not show any difference in the response to orthostasis between the phases. This is indeed surprising; we had hypothesised that the endothelial responses would be greater during the luteal phase (when estrogen levels are raised). It may be possible that the sex steroids present during the menstrual cycle do not affect the endothelial responses to orthostasis (reported above). That is, that the response works independent of any affect the sex steroids may be having on the endothelium. Nevertheless, care needs to be taken when reaching this conclusion as sample sizes were low (subject numbers were 15-17 in previous studies [24,25], and direct measurements of hormone levels in subjects were not taken in our study.

Similarly in this study we did not see any differences in the endothelial responses to orthostasis when females taking the OCP were in the placebo or the active phase. As in the case of the luteal phase of the menstrual cycle, it may have been expected that the response to orthostasis would be greater in the active phase. Interestingly, other studies looking at the effects of the OCP on the endothelium have reported widely varying and often contrasting results. A small study involving 12 females found that endothelial function, measured with brachial artery flow mediated dilatation was greater during the active phase than the placebo phase [20]. Conversely, a reduction in endothelial function during the active phase was reported in other studies [18,19]. An important distinction between these studies has been the varying proportions of estrogen and progesterone in the pill used. Meendering et al. (2010) used pills with a higher proportion of estrogen, which may explain to some extent the improved endothelial function seen. There was no difference found in the endothelial response to orthostasis between females taking the OCP (in our study group most of the females took the combined pill or pills containing only progesterone) and females having normal menstrual cycles. These results add further evidence for the limited role of sex steroids in regulating the vasodilatory endothelial response to orthostasis. Once again, however, the low sample size and the fact that hormone levels were not measured need to be considered here.

A limitation of this study is that we did not include a post-menopausal group. Despite the fact that males have lower estrogen levels than females, the breakdown of testosterone in males to estradiol means it may still be having an effect. Having a post menopausal group would have allowed study in subjects not under the effects of sex steroids, in particular estrogen. This may have better elucidated the importance of sex steroids in the endothelial response to orthostasis.

### Conclusions and future directions

This study shows that there is an increased vasodilatory endothelial response following a period of orthostasis in both females and males. The EndoPAT^®^ device could provide a less technically difficult, user-independent and mobile way to measure endothelial function in future studies.

Gender and sex hormones appear to have limited effect of the endothelial response to orthostasis. Further work including post menopausal women and blood sampling would be needed to understand the role of sex steroid hormones and their relationship to orthostatic tolerance.

Further studies need to be done to see if exposures to microgravity or bed rest induced deconditioning lead to exaggerated vasodilatory endothelial response to orthostasis. Given that the endothelial response may be modulating the sympathetic response to orthostasis, an exaggerated endothelial response may overcompensate for the sympathetic drive. Thus the endothelium may play a role in the pathophysiology of orthostatic intolerance in returning astronauts or in bedrest deconditioned subjects.
